# A Comparative Study on the Structural, Physicochemical, Release, and Antioxidant Properties of Sodium Casein and Gelatin Films Containing Sea Buckthorn Oil

**DOI:** 10.3390/polym17030320

**Published:** 2025-01-24

**Authors:** Dariusz Kowalczyk, Monika Karaś, Waldemar Kazimierczak, Tomasz Skrzypek, Adrian Wiater, Artur Bartkowiak, Monika Basiura-Cembala

**Affiliations:** 1 Department of Biochemistry and Food Chemistry, Faculty of Food Sciences and Biotechnology, University of Life Sciences in Lublin, Skromna 8, 20-704 Lublin, Poland; monika.karas@up.lublin.pl; 2 Department of Biomedicine and Environmental Research, Faculty of Medicine, John Paul II Catholic University of Lublin, Konstantynów 1J, 20-708 Lublin, Poland; waldemar.kazimierczak@kul.pl (W.K.); tomasz.skrzypek@kul.pl (T.S.); 3 Department of Industrial and Environmental Microbiology, Faculty of Biology and Biotechnology, Maria Curie-Skłodowska University, Akademicka 19, 20-033 Lublin, Poland; adrian.wiater@mail.umcs.pl; 4 Center of Bioimmobilisation and Innovative Packaging Materials, Faculty of Food Sciences and Fisheries, West Pomeranian University of Technology in Szczecin, Klemensa Janickiego 35, 71-270 Szczecin, Poland; artur-bartkowiak@zut.edu.pl; 5 Institute of Engineering Sciences, Faculty of Materials, Civil and Environmental Engineering, University of Bielsko-Biala, Willowa 2, 43-309 Bielsko-Biała, Poland; mbasiura@ubb.edu.pl

**Keywords:** edible films, sodium casein, gelatin, sea buckthorn oil, FTIR, X-ray diffraction, WVP, mechanical properties, controlled release, DPPH*

## Abstract

The aim of this study was to compare the effect of increasing concentrations (0, 1, 2, 4%) of sea buckthorn oil (SBO) on the structural, physicochemical, release, and antioxidant properties of glycerol-plasticized sodium casein (NaCAS) and gelatin (GEL) films. Ultrasonic treatment ensured effective homogenization of SBO in both types of emulsions, resulting in yellow-tinted semi-opaque films with relatively low micro-roughness. Generally, GEL films demonstrated lower UV barrier properties and solubility but exhibited higher compactness, crystallinity, transparency, surface hydrophobicity, oxygen barrier performance, strength, and antiradical activity compared to their NaCAS-based counterparts. In a concentration-dependent manner, SBO decreased the solubility and water absorption of the gelatin-based film and enhanced its oxygen permeability. Conversely, SBO improved the water vapor barrier properties of both films in a concentration-independent manner. At the highest SBO concentration, the tensile strength of NaCAS- and GEL-based films decreased by 27% and 20%, respectively, while their antiradical activity increased by 9.3× and 4.3× (based on the time required for the half-neutralization of 2,2-diphenyl-1-picrylhydrazyl radicals). Migration studies showed that at the lowest concentration, SBO was released (into 95% ethanol) approximately 2× faster from the GEL-based film than from the NaCAS film, whereas at higher concentrations, the trend reversed.

## 1. Introduction

The growing environmental concerns related to plastic packaging waste, harmful substances in plastics, and the depletion of fossil fuel resources have indeed spurred significant efforts to find sustainable alternatives, especially in the food industry. These concerns are driving innovation in packaging materials, manufacturing processes, and product design. Some companies, in addition to biodegradable materials, are exploring edible packaging made from food ingredients such as proteins, polysaccharides, and lipids. This approach not only helps reduce pollution but also allows the packaging to be consumed, potentially offering health benefits while eliminating the need for disposal. Edible packaging provides a sustainable alternative to traditional plastic, especially suitable for use in situations where plastic is restricted. Well-known examples include collagen casings for sausages and capsules or edible bags/porches (e.g., for pre-measured ingredients) that dissolve in water as the meal is cooked [1,2,3,4]. Edible packaging offers a wide range of potential functions and properties (e.g., active, smart, anti-sprouting, UV-blocking, odor/taste masking, 2D and 3D printed, etc.); however, it also presents inherent limitations (e.g., inadequate mechanical strength and sealing performance, scaling-up challenges, poor water vapor and oxygen barrier properties, lack of toxicological studies, and insufficient legal considerations), which may vary depending on the composition of raw materials [5].

Despite the current boom in plant-based foods and materials, animal-origin biopolymers (e.g., chitin, collagen, milk proteins, fibroin, elastin, keratin, resilin, worm cement proteins, mussel adhesive proteins) possess unique and irreplaceable properties, with some being particularly valuable for the development of modern eco-friendly packaging [6,7]. In comparison to polysaccharides, proteins offer greater versatility due to the presence of 20 polar and non-polar amino acids, whose varying proportions enable the tailoring of their properties for a wide range of uses and functions. Casein (CAS) and gelatin (GEL) are two contrasting proteins in terms of their amino acid composition, which contribute to their distinct functional properties in food and industrial applications.

CAS, a major protein of milk, can be processed into biodegradable plastic with good mechanical strength and durability. It is worth mentioning that CAS has a long tradition of use as a functional biomaterial. In the early 20th century, before synthetic plastics became widespread, CAS-based plastic (known as Galalith or Erinoid), made from CAS and formaldehyde, was used for items such as buttons and combs. Production was generally discontinued in the 1980s [8], but some companies are reviving CAS plastic manufacturing, now using only natural ingredients without chemical transformation [9]. CAS types available on the market include acid CAS, rennet CAS, micellar CAS, sodium caseinate (NaCAS), calcium caseinate, and hydrolyzed CAS, each serving specific applications in food, nutrition, and industrial products. When mixed with water and a plasticizer, some of them exhibit good film-forming properties, enabling the creation of transparent, flexible, and moderately strong films that can serve as effective barriers to gases (O_2_ and CO_2_), which is essential for food preservation. These films dissolve nearly instantaneously in water [10,11,12], which could be either a drawback or desirable, depending on the specific application.

GEL is a highly versatile biopolymer widely used in edible packaging due to its excellent film-forming properties, melting at body temperature, thermo-reversibility, transparency, flexibility (after plasticization), and ability to act as an effective barrier to oxygen [13,14]. Among various biopolymer-based films, GEL films are exceptionally strong [15,16], and in addition, they can be easily thermally welded [17], making them ideal for preserving food quality and extending shelf life. A prime example of this is the use of soft and hard GEL capsules in the pharmaceutical and nutraceutical industries [14,18]. It is worth mentioning that the use of GEL, which is derived from animal collagen-containing by-products such as bones and skin, aligns with the principles of the circular economy by repurposing waste materials and reducing the overall environmental impact [13].

Unfortunately, most protein-based films (as well as polysaccharide-based films) degrade rapidly in humid environments due to their hydrophilic properties, which are exacerbated by the presence of hydrophilic plasticizers, making them less effective as moisture barriers compared to synthetic alternatives. A range of strategies focused on enhancing biopolymer properties have been developed [5]. An easy and effective approach to improve their moisture resistance is hydrophobization with waxes or oils, especially in the case of proteins, whose amphipathic character can aid in emulsifying and stabilizing film-forming formulations. This provides proteins with an advantage over most polysaccharides. Moreover, protein-based films can exhibit stronger biological properties, including antioxidant potential, which further enhance their advantages over polysaccharide-based films [19,20].

Oil from sea buckthorn (*Hippophae rhamnoides* L.) fruit is known for its rich composition of essential and unsaturated fatty acids, including ω-3 (α-linolenic acid), ω-6 (linoleic acid), ω-7 (palmitoleic and vaccenic acids), and ω-9 (oleic acid). Notably, the ω-7 fatty acids content in sea buckthorn oil (SBO) is higher than that of any other plant source. Additionally, SBO is abundant in carotenoids (up to 0.5%), vitamin E, phytosterols, and trace elements. These compounds contribute to its distinct properties, offering various health benefits such as anti-inflammatory and neuroprotective effects, alleviating dry eyes, and supporting skin and overall health. Therefore, SBO is offered as a dietary supplement or used as a component in cosmetics [21,22,23,24,25]. Because of its high levels of carotenoids, SBO is a promising natural compound for photo-protection [26]. Some authors [27] have also successfully used SBO to increase the oxidative stability and, consequently, the shelf life of vegetable oils. The SBO-enriched oils acquired new sensory qualities, such as the color derived from carotenoids, as well as health-promoting properties, including improved antiradical activity. It should be noted that SBO is a drying oil, meaning it has the ability to dry and harden at room temperature, making it an effective binder. SBO’s properties and nutraceutical potential can enhance protein-based films’ water affinity, meeting the demand for healthier food packaging. It should be noted that SBO, being rich in unsaturated fatty acids, is prone to oxidation; however, microencapsulation technology effectively reduces the risk of rancidity [28]. Incorporating SBO into a protein matrix may also offer protective effects, as protein molecules like CAS and GEL in emulsions create a physical barrier at the oil-water interface [29,30]. Although the properties of films containing SBO have not yet been extensively studied, the academic community has started exploring the potential of sea buckthorn for sustainable packaging design. For instance, it has been found that starch films incorporated with sea buckthorn pomace extract improve the storage quality of beef jerky [31].

The aim of this study was to compare the effect of increasing concentrations of SBO (0, 1, 2, and 4% *w*/*w*) on the structural, physicochemical, release, and antioxidant properties of edible films based on NaCAS and GEL. By understanding the differences between these two materials, this research offers insights into optimizing film formulations to meet diverse applicational needs.

## 2. Materials and Methods

### 2.1. Materials

NaCAS (Kazeina Polska Sp.z o. o., Pułtusk, Poland), type A GEL with Bloom strength of 240 (Kamis, McCormick Polska S.A., Stefanowo, Poland), and SBO (fat content 97,3%; Altermedica, Żywiec, Poland) were used as film-formers. Glycerol (plasticizer) was purchased from Sigma-Aldrich (Burlington, MA, USA).

### 2.2. Preparation of Films

The films were prepared from film-forming solutions (FFSs) comprising NaCAS or GEL (10% *w*/*w*), glycerol (4% *w*/*w*), and SBO (1, 2, or 4% *w*/*w*). The preparation process began with mixing the protein, water, and glycerol, followed by heating the mixture in a water bath at 90 °C for 30 min under constant stirring. Once cooled to approximately 30 °C, the FFSs were combined with SBO using a Tefic 1800W probe sonicator equipped with a 20 mm diameter probe (TEFIC BIOTECH CO., Ltd., Xi’an, China) at 80% ultrasonic power. The homogenization process was conducted in a 2 s on, 2 s off cycle for two intervals of 1 min each, with a 30 s pause between intervals. As a result of ultrasound treatment, the temperature of the FFSs increased to a maximum of 70 °C. The FFSs were degassed using a stainless steel tea strainer mesh sieve, cast onto trays with areas of either 144 cm^2^ or 4 cm^2^ (depending on the test), and dried at ~25 °C with 50% relative humidity (RH) for 24 h. To ensure consistent film thickness (100 ± 10 μm), a constant mass of non-water ingredients (0.0125 g/cm^2^) was cast onto the trays. FFSs without SBO served as the controls.

### 2.3. Thickness and Conditioning

The films were cut into samples, and their thickness was measured using a Mitutoyo 547-401 digital thickness gauge (Mitutoyo, Tokyo, Japan). Subsequently, the film samples were conditioned in an MLR-350 climatic test chamber (Sanyo Electric Biomedical Co., Ltd., Osaka, Japan) for 48 h at 25 °C and 50% RH.

### 2.4. Microstructure Analysis

The FFSs were observed using an inverted CKX53 microscope (Olympus, Tokyo, Japan). The air-exposed surface of the films (during the drying process) and their cross-section (prepared by immersing the films in liquid nitrogen and fracturing) were analyzed using a LEICA 5500B microscope (Leica Microsystems GmbH, Wetzlar, Germany) equipped with a differential interference contrast (DIC) optical system. The 2D images were transformed into 3D topographies using ImageJ 1.54j software to determine the film’s air-side surface roughness, measured as root-mean-square roughness (Rq) [32]. Moreover, the air-side surface and cross-section of the films were analyzed using scanning electron microscopy (SEM) (Carl Zeiss Ultra Plus, Oberkochen, Germany). Prior to SEM observation, the film samples were coated with a thin layer of gold. The SEM analysis was conducted using an accelerating voltage of 3 or 5 kV, a working distance of approximately 12 mm, and a secondary electron detector.

### 2.5. Attenuated Total Reflectance Fourier Transform Infrared Spectroscopy (ATR/FTIR)

The FTIR spectra of the films were recorded using an FTIR spectrometer (Perkin Elmer Spectrophotometer 100, Waltham, MA, USA) operated in ATR mode. Spectra were acquired over the range of 4000–650 cm⁻^1^, with 100 co-added scans at a resolution of 4 cm⁻^1^. The collected spectra were normalized, baseline-corrected, and analyzed using SPECTRUM v10 software (Perkin Elmer, Waltham, MA, USA). Each film type was scanned twice, demonstrating good reproducibility. For better interpretation of the results, the ATR/FTIR spectrum of SBO was also obtained.

### 2.6. Wide-Angle X-Ray Diffraction (WAXD)

X-ray diffraction patterns for control and 4% SBO-enriched films were recorded using a URD 6 Seifert X-ray diffractometer (FPM-Seifert, Freiberg, Germany) with a Cu Kα radiation source. The measurement settings included a current of 30 mA, a voltage of 40 kV, and a 2θ range from 2° to 50°, with a step size of 0.1° and a scanning rate of 0.1° per 15 s at approximately 25 °C. Duplicate scans were performed for each sample, ensuring high reproducibility.

### 2.7. Optical Properties

The light-barrier properties of the film samples (1 × 4 cm) were assessed using a spectrophotometer (Lambda 40, Perkin–Elmer, Shelton, CT, USA) at wavelengths ranging from 200 to 700 nm. The spectrophotometer was calibrated with air. The film sample was then placed in the designated slit, where a cuvette is normally positioned, and a UV/Vis spectrum scan was performed. The opacity of the films was calculated using Equation (1)

(1) $$Opacity=\frac{A_{600}}{t}$$

where A_600_ is the absorbance of the film sample at 600 nm and t is the film sample thickness (mm).

Yellowness index (YI) was calculated by Equation (2)

(2) $$YI=\frac{142.86\;b^{*}}{L^{*}}$$

where b^∗^ represents the yellow/blue coordinate and L^∗^ denotes lightness. These parameters were measured using a colorimeter (NH310, 3nh, Guangzhou, China) against a white background with the reference values L* = 88.4, a* = 2.2, and b* = 2.0.

The optical analyses were conducted five times.

### 2.8. Water Affinities

The film specimens (2 × 2 cm), weighed to the nearest 0.001 g, were dried in an oven at 105 °C for 24 h. The moisture content (MC) was calculated as the percentage of water lost from the samples during drying. Film samples (2 × 2 cm) were immersed in 30 mL of distilled water for 1, 2, 5, 10, 20, 30, and 60 min at 22 °C. Subsequently, the films in their swollen state were gently blotted with tissue and reweighed. The degree of swelling (Sw) was calculated as the percentage increase in the film’s mass. The remaining samples were gently transferred into pre-weighed dishes and conditioned at 25 °C and 50% RH for 48 h. Solubility (So) was calculated as the percentage of the film dissolved in water. The analyses for MC, Sw, and So were performed in quadruplicate.

Water contact angle (WCA), an indirect indicator of hydrophilicity/hydrophobicity of the film’s surface, was determined using a DSA100B drop shape analyzer (KRÜSS GmbH, Hamburg, Germany). A 20 µL drop of deionized water was automatically dispensed onto the film surface, and the angle between the water droplet and the film surface was determined. The measurements were conducted in 6 repetitions.

WVP (g mm m^−2^ day^−1^ kPa^−1^) was calculated as follows:

(3) $$WVP=\frac{WVTR\times t}{{∆}_{p}}$$

where WVTR is the water vapor transmission rate (g m^−2^ day^−1^) measured gravimetrically based on the ISO 2528 method [33], t is the mean film thickness (mm), and Δ_p_ is the difference in the water vapor pressure (kPa) between two sides of the film.

In brief, poly (methyl methacrylate) permeation cell cups with an internal diameter of 7.98 cm (exposed film area = 50 cm^2^) and an internal depth of 2 cm were filled with 30 mL of distilled water. Film samples with a diameter of 10 cm were placed over the circular openings and sealed with O-ring rubber gaskets and screw tops. The cups were then placed in the test chamber at 25 °C and 50% RH. Weight loss was monitored over 12 h, with measurements taken every 2 h. The slopes of the steady-state (linear) sections of the weight loss versus time curves were used to calculate the WVTR. The WVP analyses were performed in triplicate.

### 2.9. Oxygen Permeability (O_2_P)

O_2_P (cm^3^ μm)/(m^2^ day) was measured using a PermeO_2_ instrument (Extra Solution Instrument, Lucca, Italy) at 23 °C and 30–50 ± 3% RH with permeability area of 50 cm^2^. The test gas was oxygen (purity ≥ 99.95%), while the carrier gas was nitrogen (purity 5.0) doped with 1% hydrogen. Measurements were conducted until recording a steady line for the oxygen transmission rate. The O_2_P analyses were performed in triplicate.

### 2.10. Mechanical Properties

The mechanical properties of the films in six repetitions were determined using a TA-XT2i texture analyzer equipped with a 50 kg load cell (Stable Micro Systems, Godalming, UK). To perform the tensile test, the initial grip separation was set to 30 mm, and the film samples (2 × 5 cm) were stretched at a speed of 1 mm s^−1^. Tensile strength (TS, MPa), elongation at break (EB, %), and elastic modulus (EM, MPa) were calculated using commonly known equations [32].

### 2.11. SBO Release and Mathematical Modeling

The film sample (4 cm^2^) was transferred into a custom-made release-measurement device (consisting of a test tube coupled with a cuvette; Figure S1) containing 5 mL of 95% ethanol. The assembled devices were shaken using an ES-60 incubator (MIULAB, Hangzhou, China) at 140 rpm at 22 °C. The absorbance of the acceptor solution was measured periodically over 24 h at 450 nm using a UV-1280 spectrophotometer (Shimadzu, Kioto, Japan). Different concentrations of SBO in ethanol were prepared and their absorbance was measured. The SBO content (mg/mL) in the acceptor solution was then estimated based on the obtained standard curve. The SBO release results were expressed as the percentage of SBO released from the films. Based on calculations, the initial SBO contents in the film samples were 3.3 mg/4 cm^2^, 6.2 mg/4 cm^2^, and 11.2 mg/4 cm^2^. The release studies were conducted in triplicate.

The SBO release kinetics were modeled using DDSolver 1.0, an add-in software for Microsoft Excel [34]. Seventeen mathematical models were tested to evaluate the data fit (Data S1). The values of the adjusted coefficient of determination (R^2^_adj_) were used to select the optimal model, based on which the times required for 50% SBO release (t_50%_) were estimated.

### 2.12. Antioxidant Activity

The antiradical activity of the films was determined using a 0.1 mM 1,1-diphenyl-2-picrylhydrazyl radical (DPPH*) solution. The film sample (4 cm^2^) was transferred into the aforementioned custom-made device (Figure S1) containing 5 mL of the DPPH* solution. The assembled devices were shaken using an ES-60 incubator (MIULAB, Hangzhou, China) at 140 rpm at 22 °C. The absorbance of the DPPH* solution was measured periodically over 20 h at 515 nm using the spectrophotometer. The affinity of the films to quench DPPH* (%) was calculated in percentage using Equation (4):

DPPH* scavenging = [(A_0_ − A_1_)A_0_] × 100 (4)

where A_0_ is the absorbance of the DPPH* solution before the test and A_1_ is the absorbance of the DPPH* solution after contact with the film sample.

The tests were performed in triplicate. Mathematical modeling of the kinetics of antiradical activity was conducted (Data S2) as described in Section 2.11 in order to estimate the time required to scavenge 50% of DPPH* (t_DPPH*50%_).

### 2.13. Statistical Analysis

Statistical differences between mean values were assessed for significance at the level of *p* < 0.05 using analysis of variance followed by Fisher’s post hoc test, performed with STATISTICA 13.3 software (StatSoft Inc., Tulsa, OK, USA).

## 3. Results and Discussion

### 3.1. Microstructure

**Figure 1:**
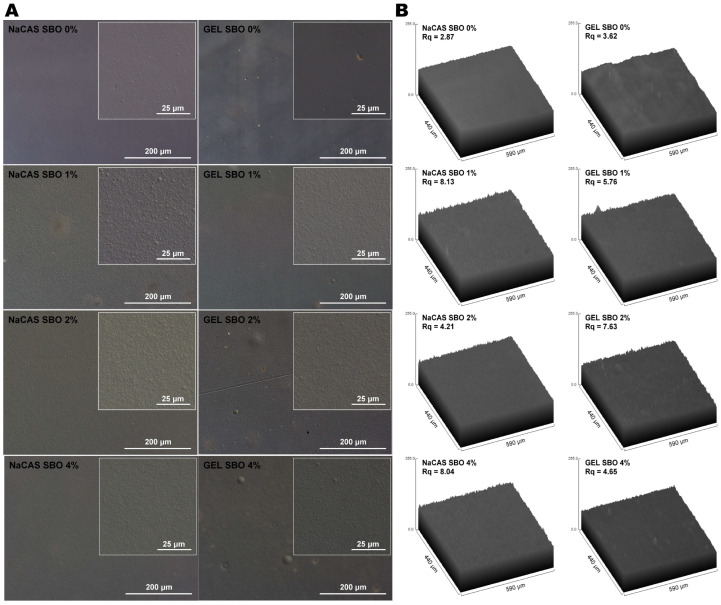
200× DIC microscopy images (**A**) and 3D representations with calculated root-mean-square roughness (Rq) (**B**) of the air-side surface of films made from sodium caseinate (NaCAS) and gelatin (GEL) with increasing concentrations of sea buckthorn oil (SBO).

**Figure 2:**
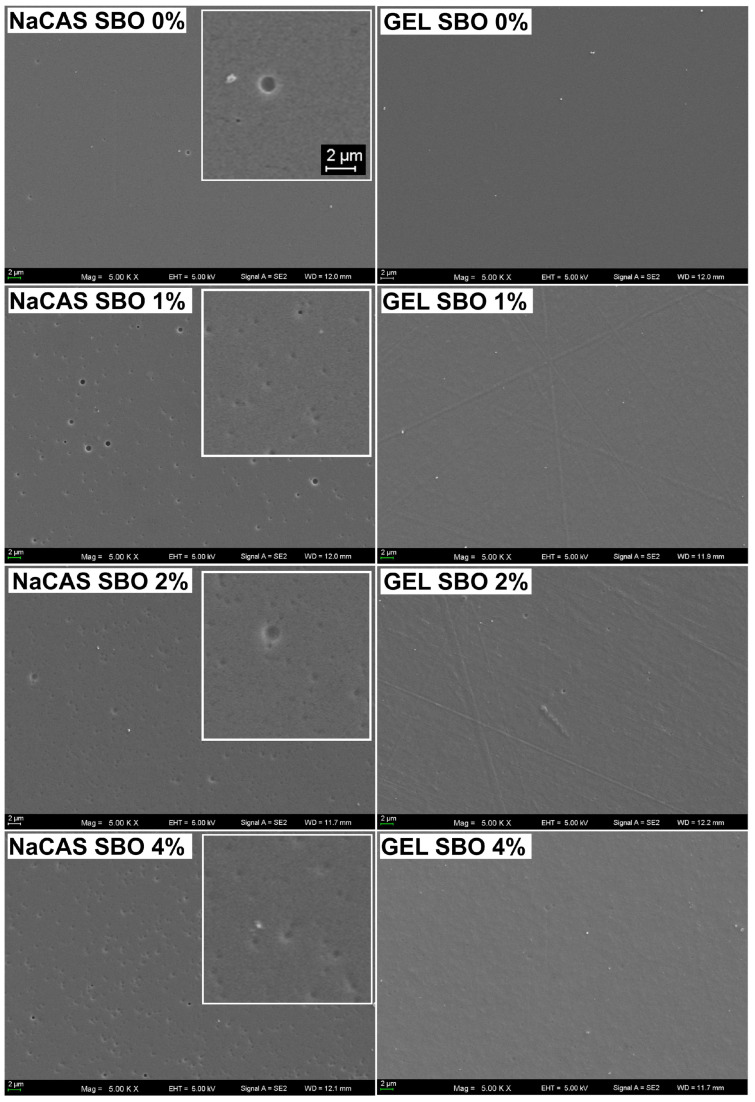
5000× SEM images of the air-side surface of films made from sodium caseinate (NaCAS) and gelatin (GEL) with increasing concentrations of sea buckthorn oil (SBO).

**Figure 3:**
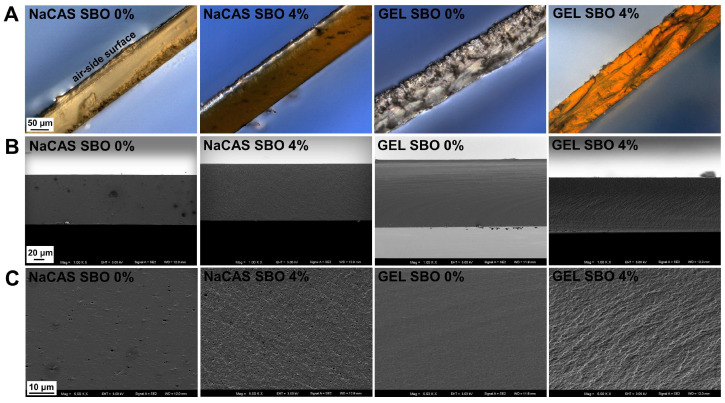
Cryo-fractured cross-sections of films made from sodium caseinate (NaCAS) and gelatin (GEL), without and with addition of sea buckthorn oil (SBO), visualized by differential interference contrast microscopy at a magnification of 200× (**A**) and scanning electron microscopy at magnifications of 1000× (**B**) and 5000× (**C**).

Microscopic observations of the FFSs revealed excellent oil emulsification, as hardly any oil spheres were visible (Figure S2). This indicates that the combined action of the amphiphilic nature of the proteins and ultrasonic emulsification generated kinetically stable FFSs. As demonstrated by DIC microscopy, the surface of the NaCAS film was covered with numerous spherical microparticles (<1 µm, Figure 1), which may represent fat globules present in the NaCAS. According to the manufacturer’s information, the fat content in NaCAS was ≤2%. Interestingly, these particles were not observed in the SEM images (Figure 2). At the microscopic level, the surface of the emulsion-based films was rougher compared to that of the control films (*p* < 0.05), as clearly indicated by a comparison of the Rq values (Figure 1). Larger oil droplets were more frequently observed in the GEL-based films than in the NaCAS-based ones, indicating that GEL provided slightly weaker dispersion and/or stability of SBO droplets. This is somewhat surprising, as it could be expected that the solidification of GEL-based emulsions during casting would effectively prevent emulsion breakdown caused by flocculation or coalescence [15]. Despite this, the literature indicates that NaCAS exhibits superior emulsifying properties compared to GEL. Due to its lower surface activity at the oil–water interface, GEL forms emulsions with larger oil droplets that cream rapidly and are less stable toward droplet coalescence [35]. Despite the high magnification, SEM images offered less detailed information about the oil distribution in the films compared to DIC microscopy (Figure 1 and Figure 2). However, unlike DIC microscopy, SEM enabled the visualization of the porous microstructure in the NaSC films (Figure 2 and Figure 3). This could result from holes caused by air bubbles, suggesting that more attention should be given to degassing NaCAS-containing formulations. In line with the present results, previous studies [36] have also shown that the ability of CAS to decrease the surface tension of water (which, as is well known, makes it an excellent whipping and emulsifying agent) promotes air bubble incorporation and the formation of large holes in CAS/GEL beads. This, in turn, affected their properties, such as drug loading, drug release, and flotation. The SEM observations of cryo-fractured surfaces revealed that, unlike NaCAS, GEL produced films with a compact microstructure (Figure 3). The presence of SBO resulted in a less smooth fracture surface, indicating a heterogeneous structure of the emulsion films.

### 3.2. ATR/FTIR

#### 3.2.1. ATR/FTIR of SBO

**Figure 4:**
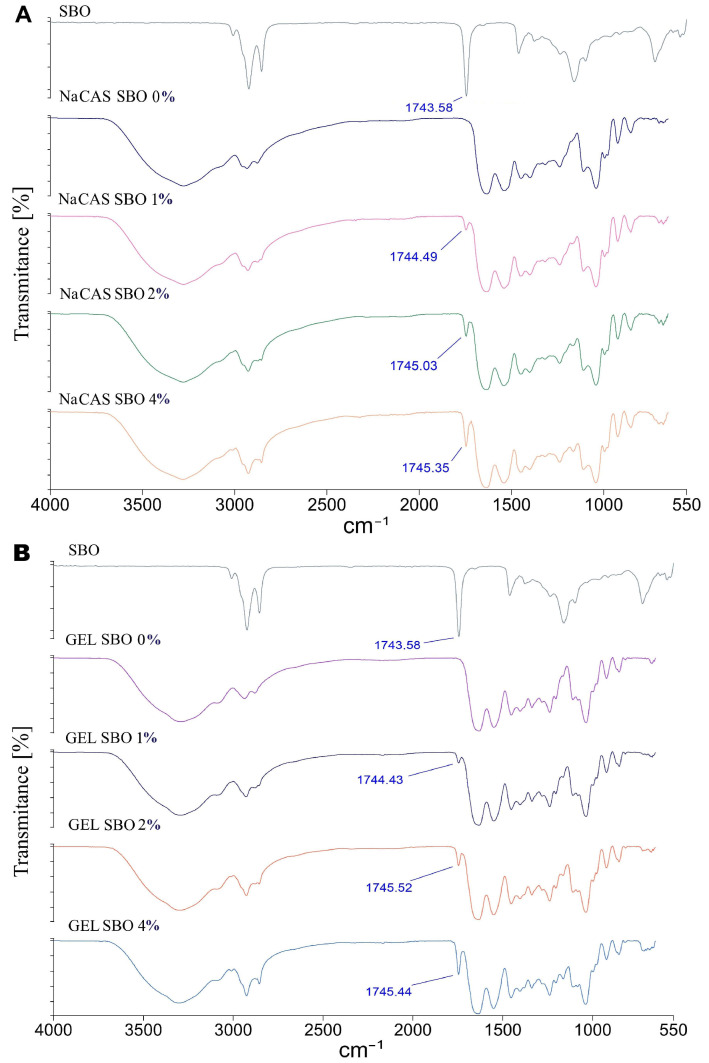
ATR/FTIR spectra of sea buckthorn oil (SBO) and films made from sodium caseinate (NaCAS) (**A**) and gelatin (GEL) (**B**) with increasing concentrations of SBO.

The vibration bands typical for triacylglycerols were observed in the ATR/FTIR spectra of SBO, including (i) stretching of =C-H (trans and cis) bonding at 3009 cm⁻^1^, (ii) the asymmetric and symmetric stretching vibrations of -C-H (methyl (CH_3_) and methylene(CH_2_) groups) at 2854 cm⁻^1^ and 2923 cm⁻^1^, respectively, and (iii) the C=O stretching vibrations of the carboxyl and ester group (R-COO-R′) at 1744 cm⁻^1^ (Figure 4). At 1460 cm⁻^1^, a band corresponding to the CH_2_ scissors deformation vibration was detected, while the bands at 1098 cm⁻^1^ and 1106 cm⁻^1^ were attributed to the vibration of C-O ester and CH_2_ groups. Generally, the spectrum of SBO shows a very similar pattern to those reported for other oils, which can be explained by the close chemical structure of the fatty acid residues. However, compared to the spectral characteristics of typical vegetable oils, SBO displays additional bands in the 900–1250 cm⁻^1^ region, which are attributed to carotenoids [37,38].

#### 3.2.2. ATR/FTIR of the Films

The spectra of all films exhibited a broad amide A peak, located at approximately 3289 cm⁻^1^ for the GEL film and 3277 cm⁻^1^ for the NaCAS film (Figure 4), which can be attributed to the –OH and –NH_2_ groups of the protein, as well as the –OH group from glycerol and water. This band slightly shifted to higher or lower wavenumbers as a result of SBO incorporation. Since the amide A band depends on the strength of the hydrogen bonding of the NH group, the observed changes suggest that SBO may have a significant effect on the self-interaction of protein functional groups or the glycerol–protein interaction by hydrogen bond formation. Compared to the controls, the emulsion-based films displayed slightly higher peaks at around 2920 cm^−1^ and 2860 cm^−1^, which can be attributed to the presence of hydrocarbon chains in the SBO, giving intense stretching (C-H) vibrations from the CH_2_ and CH_3_ groups, as mentioned in Section 3.2.1. The intensity of the peak at ~1744 cm⁻^1^ increased notably with higher SBO content. This peak, absent in the spectra of both control films, proved to be the most reliable marker for SBO presence. A similar observation was made for the GEL-based film incorporated with olive oil [39]. However, it is not always the rule that a peak at ~1744 cm⁻^1^ is visible in protein–lipid films [40].

All film spectra displayed characteristic amide I (1630 cm⁻^1^), amide II (1540 cm⁻^1^), and amide III bands (~1240 cm⁻^1^), which are associated with the peptide bond. With the addition of SBO, these peaks exhibited a slight shift toward higher wavenumbers (Figure 4), potentially reflecting some changes in the protein’s secondary structure [41].

### 3.3. WAXD

**Figure 5:**
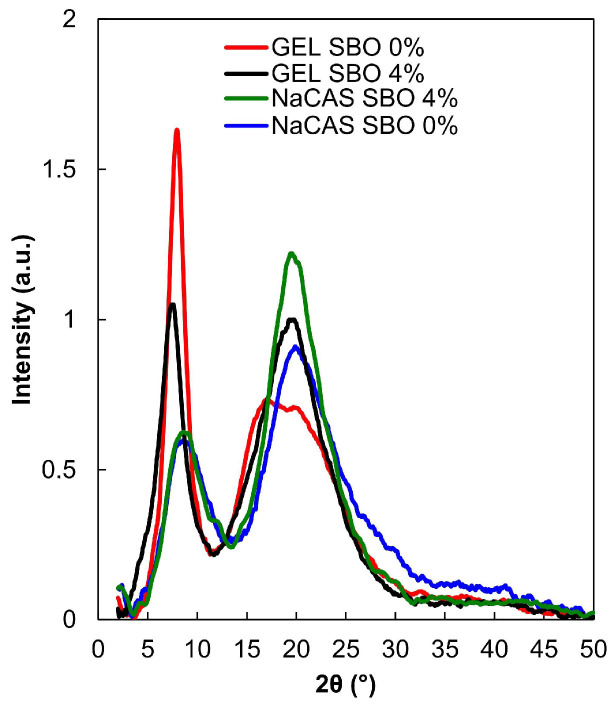
WAXD patterns of films made from sodium caseinate (NaCAS) and gelatin (GEL), without and with addition of sea buckthorn oil (SBO).

It is well known that polymer chains that are arranged in a regular, periodic manner with strong interactions form crystalline regions. In turn, polymer chains that are arranged irregularly and have less density (present more free volumes compared to crystalline regions) are amorphous [42]. Both NaCAS- and GEL-based films had a semi-crystalline structure with two diffraction peaks (Figure 5). Consistent with the earlier observations [16,19,43], the GEL films showed a strong narrow peak located at 2θ ≈  7.9° and a broad peak located at 2θ ≈ 20°. The first peak signifies the reconstruction of the triple-helical collagen crystalline structure, whereas the second band is associated with the spacing of amino acid residues along the helix, corresponding to the amorphous portion of GEL [19]. The NaCAS films exhibited peaks at 2θ  =  8.6° and 2θ ≈ 20°, which is also in agreement with the literature [44]. The sharp peak observed in the diffractograms of the NaCAS-based films might be attributed to the heat treatment, cooling, and drying, which promoted some level of ordering or local crystallinity [45]. The GEL films had a higher intensity of the first diffraction peak (the content of crystalline fraction) compared to the NaCAS-based films (Figure 5). This outcome can be attributed to the distinct amino acid composition of GEL, specifically the repetitive triplets of glycine, proline, and hydroxyproline, which promote a high degree of super-organization in the protein chains [43]. The more complex amino acid and fraction profile of CAS (a mixture of αS1-, αS2-, β-, and κ-caseins) [46] likely did not promote the formation of a highly self-organized crystalline domain.

The presence of SBO affected the semi-crystalline structure of the films (Figure 5). Regardless of the film type, a significant increase in the intensity of the second band was observed, which could be attributed to the lower protein content in the FFS films cast onto the plates due to the presence of a significant amount of SBO in the formulation. In the case of GEL films, a significant decrease in the intensity of the first crystalline peak was observed. In addition to the above-mentioned reason, this could be caused by the oil phase, which likely weakened protein-protein interactions, limiting the rearrangement of the triple-helical structure in GEL films. This assumption appears to be supported by the fact that in NaCAS films, SBO did not provoke a decrease in the intensity of the first peak, likely due to the lower efficiency of CAS in self-organization, making it less sensitive to self-assembly disruptors.

### 3.4. Optical Properties

**Figure 6:**
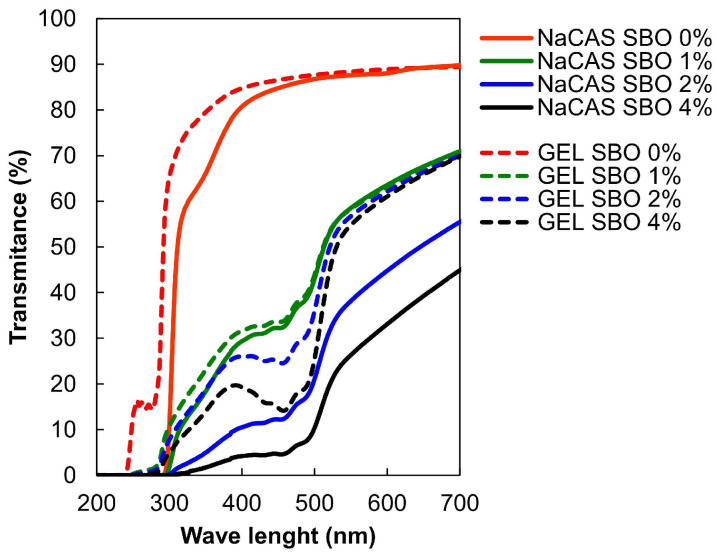
Effect of increasing sea buckthorn oil (SBO) concentrations on UV/VIS light transmission of sodium caseinate (NaCAS) and gelatin (GEL) films.

In agreement with previous studies [47], NaCS produced films with significantly better UV-blocking properties than the GEL (Figure 6). This can be attributed to the higher content of aromatic amino acids in CAS, which are known as strong UV-absorbing chromophores [48]. Consistent with the literature [49], in the spectra of the control GEL film, a distinct transmission peak was observed at ~256 nm (Figure 6), caused by light absorption around ~280 nm (Figure S3) due to the small content of aromatic side chains [50]. This peak was absent in the transmittance spectrum of the NaCS film (Figure 6), due to a higher concentration of aromatic chromophores, resulting in stronger UVC light absorption (Figure S3), thus minimal transmission. As the SBO concentration increased, the films exhibited stronger UV/VIS light blocking. Notably, an increasingly stronger β-carotene absorbance band around ~450 nm [38] was observed. Given the weaker UV absorption properties of the GEL film (Figure S3), the addition of SBO significantly enhanced its UV barrier properties (Figure 6).

The control films did not differ in terms of transparency (Table 1). This result contrasts with the findings of Bonilla and Sobral [47], who reported that NaCAS produced a more opaque film than pigskin GEL. The observed discrepancy may be attributed to variations in the purity and quality of the raw materials used. Additionally, the inconsistency could stem from differences in the preparation of the FFSs; for example, more intensive heating (90 °C vs. 55 °C) and ultrasonic emulsification (applied in our study) could favor the dissolution of the proteins [51], resulting in optically clear materials. Supporting this, a previous study demonstrated that ultrasonic treatment of CAS led to the formation of soluble aggregates smaller than the original CAS micelles [52]. Smaller particles will likely absorb or scatter less light. Due to a rougher microstructure (Figure 1 and Figure 3), the SBO-added films were more opaque than the control films (Table 1). Interestingly, increasing the concentration of SBO gradually reduced the transparency of the NaCAS film, but not the GEL film. As known, microstructural defects such as residual pores act as light scattering centers [53]. Therefore, the more opaque appearance of the NaCAS films (Table 1) can be attributed to their porosity (Figure 2 and Figure 3). Presumably, the increase in SBO content resulted in greater viscosity of the emulsions, which hindered degassing and trapped more air bubbles after casting.

The CAS- and GEL-based films exhibited similar YI, regardless of the SBO concentration (Table 1). Of course, films with higher SBO concentrations were more yellow.

### 3.5. Water Affinities

**Figure 7:**
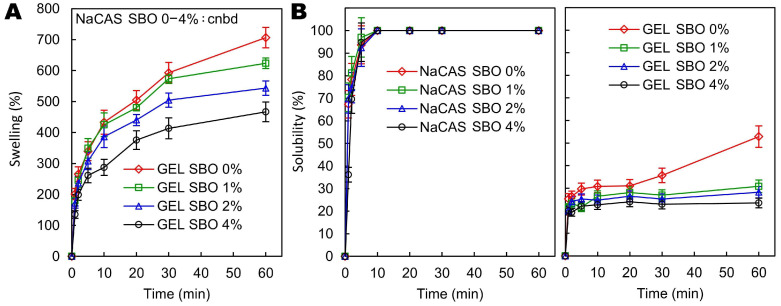
Effect of increasing sea buckthorn oil (SBO) concentrations on swelling (**A**) and water solubility kinetics (**B**) of sodium caseinate (NaCAS) and gelatin (GEL) films. cnbd—cannot be determined.

Although GEL is a hydrophilic substance, at the same time, non-polar amino acid residues make up at least 2/3 of their total number [54]. So, GEL generally has a higher level of hydrophobic amino acids compared to CAS [54,55]. Due to its unusual amino acid profile, the structure of GEL is more organized and compact than that of CAS [56] (Figure 3 and Figure 5). This tighter molecular arrangement likely reduces the number of available sites for water interaction, leading to lower MC, WCA, and So values for the GEL-based films compared to their NaCAS-based counterparts (Table 1, Figure 7). Additionally, for the control films, the GEL-based film also exhibited lower permeability to water vapor (Table 1).

The incorporation of SBO, regardless of its concentration, did not affect the MC of the films (Table 1). All NaCAS-based films, after immersion in water, were too slimy to remove the excess water, making it impossible to determine their Sw kinetics (Figure 7). Although SBO made the NaCAS film more hydrophobic (i.e., increased WCA) (Table 1), it did not reduce its So (Figure 7). This confirms that doping a highly soluble material with lipids is an ineffective way to improve its resistance to water [15]. The GEL films were partially soluble in water (So_1h_ = 23.42–52.84, depending on the SBO content) and exhibited high swelling capacity (Sw_1h_ = 466.67–706.37%, Figure 7), which aligns with the results of previous studies [16,43,57]. The increase in SBO content gradually reduced both the So and Sw of the GEL film, to levels of ~42–56% and ~12–34%, respectively, depending on the SBO content. This improvement in water resistance, however, was not accompanied by the expected increase in the hydrophobicity of the GEL film surface (Table 1). In fact, SBO slightly increased the wettability (i.e., decreased WCA) of the GEL film. In accordance with the present results, previous studies on GEL films have demonstrated that the addition of a hydrophobic plasticizer (acetyl tributyl citrate) also decreased the WCA [58]. The fact that the hydrophobicity of the SBO was an important factor for the increase in WCA of NaCAS film but not GEL film shows that, due to the different structural and physicochemical properties of these proteins, the effect of SBO on the film’s wettability cannot be generalized and remains a complex issue.

The ineffective hydrophobizing effect of SBO on GEL-based films may result from the gelling properties of GEL. Surface tension is known to influence the spreading of a liquid on a solid surface. The lower the surface tension of a material (e.g., the film surface), the more the liquid “spreads” across it, leading to a smaller contact angle (higher wettability). Proteins, being surface-active molecules, decrease the surface tension of water as they adsorb at the air–water interface. When the surface tension decreases to a certain minimum value, hydrophobic segments of the protein molecules tend to associate [59]. These domains could also interact with lipids, which may occur at the interface between the hydrophobic air and the hydrophilic aqueous solution. As a result, a hydrophobic interior could form [60], making the protein molecules more hydrophilic on the surface. Due to the solidification of the GEL solution, this state could be stabilized, leading to increased wettability of the film (Table 1). In the case of NaCAS molecules, being more hydrophilic, they could desorb back into the solution, thereby increasing the surface tension of the solution and, consequently, the film.

Likely due to the higher hydrophobic nature of the GEL-based film, as indicated by its higher WCA value (Table 1), it exhibited lower WVP than the NaCAS film (*p* < 0.05, Table 1). However, this result contrasts with the findings of Bonilla and Sobral [47], who observed that glycerol-plasticized NaCAS film had a lower WVP than GEL-based film. In turn, Choi et al. [61] found that the WVP values of GEL- and NaCAS-based sorbitol-plasticized films were similar. This inconsistency may be related, among other factors, to differences in the film preparation procedure, as discussed in Section 3.4. As demonstrated, both the heating temperature of the FFS and ultrasonic waves affect the WVP of protein-based films [62,63,64]. For example, it has been demonstrated that higher heating temperatures generally lead to a decrease in the WVP of GEL films. This is likely due to the exposure of the hydrophobic domains of the GEL chains and the more regular arrangement of the chains, which results in a more compact film matrix [62]. Unfortunately, data regarding the effect of heating on the WVP of CAS films are limited. Nevertheless, for comparison, it is worth citing results concerning other proteins. Perez-Gago et al. [65] observed that films obtained from native and heat-denatured whey protein isolate (WPI) had similar WVP. Furthermore, Guckian et al. [66] found that altering the ratio of heated to unheated protein in WPI film formulations had no effect on the WVP. Also, Kowalczyk and Baraniak [67] did not show any effect of heating (90 °C) on the WVP of films made from pea protein isolate.

SBO, regardless of concentration, significantly reduced the WVP of the NaCAS film, while 4% SBO was needed for the same effect in the GEL film (Table 1). No significant differences (*p* > 0.05) in WVP were observed among the emulsified films (Table 1). Considering the porous structure of SBO-added NaCAS films (Figure 2 and Figure 3), the WVP values suggest that the pores in these films were not continuous throughout.

### 3.6. Oxygen Permeability

**Figure 8:**
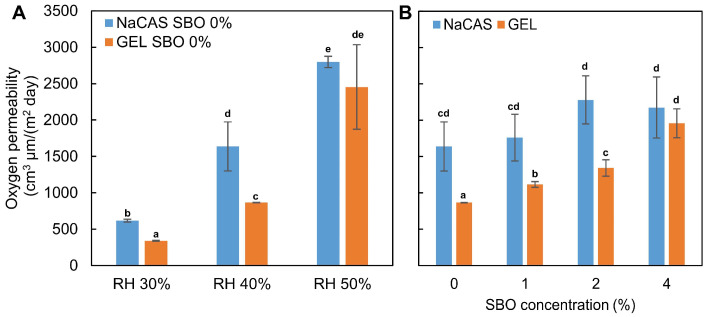
Effect of relative humidity (RH) (**A**) and increasing concentrations of sea buckthorn oil (SBO) (**B**) on the oxygen permeability of sodium caseinate (NaCAS) and gelatin (GEL) films. a–e Values with the different superscript letters are significantly different (*p* < 0.05).

Food packaging materials with high oxygen barrier properties can ensure the quality maintenance of oxygen-sensitive products throughout their shelf life and guarantee their safety. However, it should be noted that the O_2_P of polymer-based materials, especially biopolymeric ones, depends significantly on environmental conditions. As expected, based on the literature data [68], the increase in RH from 30% to 50% (the typical range of low to moderate humidity often found in air-conditioned rooms or temperate climates) resulted in a gradual increase in O_2_P of the control films (Figure 8A). Measurements of O_2_P of the 4% SBO-added NaCAS film samples under RH = 50% could not be carried out, as the O_2_ concentration in the chamber was too high (error code was generated), indicating the film (50 cm^2^) was too permeable to O_2_. Therefore, the O_2_P values obtained at RH = 40% were compared in this study (Figure 8B). Up to 2% SBO concentration, the GEL-based films were less permeable than the NaCAS-based films. In accordance with the present results, previous studies [61] have also demonstrated that the GEL film had approximately half the O_2_P compared to the NaCAS film. This could be attributed to the ability of GEL to produce materials with a very dense structure (Figure 3). Furthermore, as shown by the WAXD study (Figure 5), the GEL molecules interacted in a random or semi-ordered manner, forming more distinct crystalline films, while the structure of NaCAS-based films was more disordered (amorphous). It is believed that a polymer with a higher degree of crystallinity, compared to one with a lower degree, provides less permeation [69].

The increase in SBO gradually deteriorated the oxygen barrier properties of the GEL films (*p* < 0.05), but not those based on NaCAS (Figure 8B). In the case of the latter, an increasing trend in O_2_P was observed; however, due to large standard deviations, the data did not differ statistically significantly. The SBO-induced increase in O_2_P of the GEL films can be explained by the formation of irregularities in the ordered protein-protein structure [70]. The naturally less organized structure of the NaCAS films might have been less sensitive to the presence of these “disruptions”, as discussed in Section 3.3.

### 3.7. Mechanical Properties

As mentioned in the Introduction, among various biopolymeric film-formers, GEL produces one of the most durable materials, which is attributed to the partial renaturation of random GEL strands into the triple-helix structure found in native collagen. With strict repetition of the glycine, proline, and hydroxyproline triplets, GEL offered better self-association compared to NaCAS, leading to higher film crystallinity (Figure 5), which resulted in greater TS (12.92–16.16 MPa vs. 5.18–7.06 MPa) and EM (165.33–339.72 MPa vs. 144.11–186.38 MPa) (Table 2) [42,58]. However, surprisingly, in contrast to the results documented in previously cited works, the control NaCAS- and GEL-based films did not differ in terms of EB (Table 2).

At concentrations of 1–2%, SBO did not significantly affect the TS of the films (*p* > 0.05, Table 2), whereas at 4%, it reduced the TS of the NaCAS and GEL films by approximately 27% and 20%, respectively. This result can be attributed to the formation of significant discontinuities in the polymeric matrices, caused by a reduction in cohesive protein–protein interactions, which were overshadowed by protein–lipid and lipid–lipid attractions [40,70]. In contrast to the NaCAS film, SBO at levels of 1–2% caused a significant reduction in the EM of the GEL film (Table 2). At the maximum SBO addition level, the EM values of the NaCAS and GEL films were decreased by 22% and 51%, respectively. These findings suggest that the compact GEL network, formed through numerous ordered intra- and inter-chain hydrogen bonds [71], was more susceptible to lipid-induced disruptions, as discussed in Section 3.3. From this perspective, the lack of high self-organization in the NaCAS network rendered its rigidity less sensitive to the presence of lipids.

SBO acted as a plasticizer, increasing EB in both films, but enhanced elongation in NaCAS films was only up to 2%, after which it weakened to the initial value. SBO was more effective in plasticizing NaCAS- than GEL-based film.

### 3.8. SBO Release

**Figure 9:**
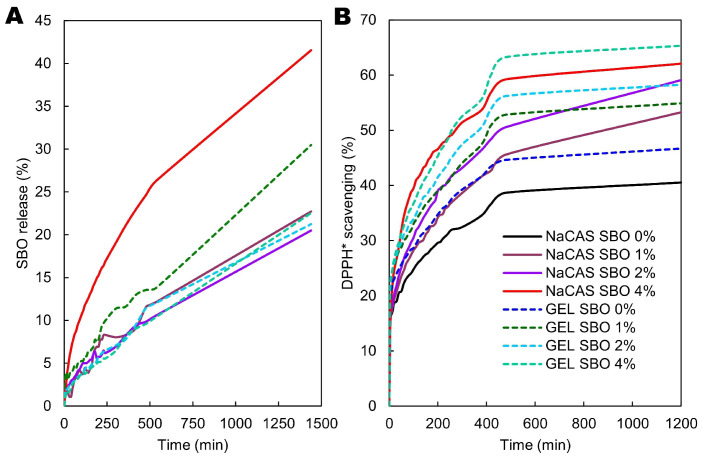
The kinetics of sea buckthorn oil (SBO) release (**A**) and antiradical activity (against DPPH*) (**B**) of sodium caseinate (NaCAS) and gelatin (GEL) films.

Figure 9A presents experimental data of the percentage cumulative release of SBO from the films into 95% ethanol—a substitute for vegetable oil (i.e., food simulant D2 assigned for foods that have a lipophilic character and are able to extract lipophilic substances [72]). Depending on the film type and SBO concentration, the release ranged from 20.49% to 30.47% during a 24 h dissolution test, without reaching a plateau within this period. Therefore, mathematical modeling was necessary to predict the t_50%_ values (Table 2). The very limited migration indicates the presence of poorly extractable SBO, likely due to the formation of lipid–protein complexes. When treated with denaturing agents, such as ethanol, the proteins, including GEL and CAS, exhibit an enhanced surface area and greater exposure of hydrophobic residues [73,74], facilitating the formation of lipoproteins with distinct buoyant densities [75]. Among the 11 mathematical models (Data S1), the Makoid–Banakar (M-B) model was the most accurate in predicting the release dynamics of the SBO from all emulsion films (mean R^2^_adj_ = 0.9844, Data S1). This confirms the rule that multi-parameter models (three or more parameters) are better at predicting release than one- or two-parameter models [32]. The M-B model is similar to the Korsmeyer–Peppas (K-P) model, with the key difference being the inclusion of an additional exponential term in its equation (Data S1). The M-B model becomes identical to the K-P model when the parameter k is zero [76], which occurred in this study (Data S1). Consequently, the K-P model accurately predicted the SBO release kinetics (mean R^2^_adj_ = 0.9774). The ‘n’ release exponent in the K-P model is used to describe the main transport mechanisms involved in the release. According to the ‘n’ value (0.5 < n < 1, Table 2), the SBO release followed anomalous transport, i.e., both diffusion of SBO and polymer relaxation (swelling/erosion) contributed to the release mechanism.

Based on the t_50%_ values, it was observed that, depending on the film-former, i.e., NaCAS vs. GEL, an increase in SBO content caused an increase and a decrease in the release rate, respectively (Table 2). The acceleration of release found for the NaCAS films can be explained by the fact that the significant increase in SBO content enhanced the concentration gradient, thereby stimulating lipid diffusion into the acceptor. Such a phenomenon has been observed, for example, in the release of hydrophobic antioxidants like ascorbyl palmitate and curcumin from polysaccharide/GEL-based films into ethanol and 50% ethanol, respectively [32,77]. It is also possible that with a higher lipid concentration, more SBO was less strongly bound to NaCAS. The increase in porosity of the NaCAS films, induced by SBO (Figure 2), could also stimulate a faster release. In contrast to the NaCAS films, the slowed SBO release (when SBO content increased from 1% to 2%) suggests that SBO affected the GEL matrix structure, making it less permeable to ethanol likely due to increased GEL-SBO complexes driven by increased hydrophobic interactions. This assumption is supported by the fact that GEL generally has a higher level of hydrophobic amino acids compared to CAS, as mentioned in Section 3.5, resulting in a greater number of possible apolar binding sites. At lower SBO concentrations, the hydrophobic interactions could be less significant, leading to a more open structure within the GEL matrix, which facilitated SBO diffusion. However, as can be seen from Table 2, increasing the SBO concentration from 2% to 4% led, as in the case of NaCAS-based films, to an accelerated release (i.e., provoked by the increasing SBO concentration gradient).

Due to the differing effects of SBO content on its release rate, it is challenging to unequivocally determine from which film SBO is released more slowly or quickly. At the lowest concentration, SBO was released ~2× faster from the GEL-based film than from the NaCAS film, whereas at the higher concentrations, the trend reversed (Table 2). As a suggestion for future research, the physicochemical properties of the films in contact with ethanol should be determined to gain a better understanding of the release mechanism.

### 3.9. Antiradical Activity

As can be seen from Figure 9B, the DPPH* scavenging process by all films can be divided into two stages: (i) an initial nearly linear rapid rate and (ii) a slow increase, not reaching a plateau. The antiradical activity of the films was quite low, with DPPH* neutralization reaching 8% to 18% after 2.5 min, while after the final 20 h test, the activity ranged from 40.51% to 65.33%, depending on the film type and SBO concentration. As a comparison, the polysaccharide/GEL films containing ascorbyl palmitate (a lipophilic derivative of vitamin C) in the amount of 1–2%, almost completely scavenged DPPH* within 3 to 65 min [77]. A note of caution is due here, as the DPPH* concentration in the cited study was much higher, making the films obtained in this study appear even less impressive in comparison. As expected, based on previous results [43,47], both control films demonstrated some potential to neutralize free radicals, which gradually increased with the rise in SBO content (Figure 9B). The comparison of the time required for the half-neutralization of free radicals showed that, in general, the GEL films exhibited significantly stronger antiradical potential than the NaCAS films, especially in the case of SBO-free samples (t_DPPH*50%_ = 1230.76 vs. 2375.67 min, Table 2). This result is linked to differences in the amino acid composition, structure, and hydrophobic characteristics of GEL and CAS. Some studies suggest that the high levels of glycine and proline in GEL contribute to its strong antioxidant properties [78]. Nevertheless, Bonilla and Sobral [47] observed that the GEL film exhibited weaker antiradical properties compared to the NaCAS film. The cause could be attributed to the different methodological approaches used. The cited authors completely dissolved the films before analysis and used the 2,2’-azino-bis(3-ethylbenzothiazoline-6-sulfonic acid) radical (ABTS*), whereas, in the present study, the film samples were in direct contact with the DPPH* solution. Partially consistent with the present results, a previous study has shown a superior antiradical activity (against ABTS*) for the GEL-based films compared to the WPI-based films [43].

The evaluation of the percentage increase in the antiradical potential for NaCAS and GEL films with 4% SBO, measured after 20 h of testing, revealed a comparable enhancement of approximately 1.5 times relative to the control films (Figure 9B). In turn, based on t_DPPH*50%,_ the presence of 4% SBO enhanced the antiradical activity of NaCAS- and GEL-based films by 9.3 times and 4.3 times, respectively (Table 2). This is a relatively satisfactory result, comparable to the addition of 0.05% fireweed extract to GEL film [43], but not as remarkable as the increase in antiradical potential observed after adding L-ascorbic acid at 0.5–2% to GEL/candelilla wax-based film [79]. It is important to note, however, that the cited studies employed ABTS*, so such a comparison is not entirely appropriate.

## 4. Conclusions

In this study, we hypothesized that the incorporation of an appropriate level of SBO could not only improve the water-resistance of the protein-based films but also enhance their antioxidant potential, without significantly compromising other properties, such as transparency and mechanical strength. The SBO-added GEL-based films have proven to be better suited for food packaging than the SBO-added NaCAS-based films due to their superior mechanical properties, better oxygen barrier performance, transparency, surface hydrophobicity, and water resistance. This was partly because GEL molecules tended to interact in a random or semi-ordered manner, leading to the formation of more defined crystalline structures, whereas NaCAS-based films exhibited a less organized (more amorphous) structure. Additionally, the GEL films exhibited higher antiradical potential, which could actively enhance food preservation by protecting against oxidative damage. However, if the application requires fast solubility in water at room temperature, NaCAS films would be an ideal solution. Both film types demonstrated nearly linear SBO migration into 95% ethanol (a fatty food simulant), suggesting they could effectively extend the shelf life of long-stored food. When the SBO-added films are used as direct contact materials for food (e.g., coatings), the majority of the antioxidants will remain on the food surface, where oxidation reactions predominantly occur. The SBO-release profiles of the films can be optimized to some extent by adjusting the carrier/SBO ratio, allowing for better control over the migration and performance characteristics. Future research could focus on investigating the long-term stability of SBO incorporated into films and testing their performance under real storage conditions for lipid-rich foods.

## Figures and Tables

**Table 1:** The effect of sea buckthorn oil (SBO) concentration on the opacity (Op), yellowness index (YI), moisture content (MC), water contact angle (WCA), and water vapor permeability (WVP) of the films based on sodium casein (NaCAS) and gelatin (GEL).

Film Type	SBO (%)	Opacity (A_600_/mm)	YI	MC (%)	WCA (o)	WVP (*)
NaCAS	0	0.63 ± 0.07 ^a^	−10.64 ± 0.26 ^a^	19.68 ± 1.14 ^d^	67.55 ± 4.05 ^a^	55.21 ± 0.99 ^bc^
	1	2.33 ± 0.14 ^b^	58.20 ± 3.80 ^b^	18.34 ± 1.49 ^cd^	88.94 ± 3.40 ^b^	54.07 ± 0.71 ^abc^
	2	3.86 ± 0.07 ^c^	87.53 ± 2.33 ^c^	19.83 ± 1.24 ^d^	88.42 ± 4.55 ^b^	51.75 ± 1.98 ^ab^
	4	5.44 ± 0.23 ^d^	120.61 ± 3.31 ^d^	18.38 ± 0.73 ^cd^	90.02 ± 5.63 ^b^	50.68 ± 1.89 ^a^
GEL	0	0.60 ± 0.13 ^a^	−7.20 ± 0.64 ^a^	16.25 ± 0.56 ^ab^	118.08 ± 3.37 ^d^	63.24 ± 0.26 ^d^
	1	2.43 ± 0.16 ^b^	52.12 ± 3.55 ^b^	17.32 ± 1.11 ^bc^	103.32 ± 2.07 ^c^	57.98 ± 4.44 ^c^
	2	2.40 ± 0.09 ^b^	92.02 ± 1.09 ^c^	16.89 ± 1.70 ^bc^	100.13 ± 2.57 ^c^	54.77 ± 4.58 ^abc^
	4	2.43 ± 0.12 ^b^	122.56 ± 2.52 ^d^	14.76 ± 1.02 ^a^	102.16 ± 1.26 ^c^	55.02 ± 1.88 ^abc^

^a–d^ Values with the different superscript letters within one column are significantly different (*p* < 0.05). * (g mm m^−2^ d^−1^ kPa^−1^).

**Table 2:** The effect of sea buckthorn oil (SBO) concentration on the tensile strength (TS), elongation at break (EB), elastic modulus (EM), time required for 50% SBO release (t_50%_), diffusional exponent (n) of the Korsmeyer-Peppas model, and time required to scavenge 50% of DPPH* (t_DPPH*50%_) of the films based on sodium casein (NaCAS) and gelatin (GEL).

Film Type	SBO (%)	TS (MPa)	EB (%)	EM (MPa)	t_50%_ (min)	n	t_DPPH*50%_ (min)
NaCAS	0	7.06 ± 1.20 ^b^	73.64 ± 22.48 ^ab^	184.40 ± 39.37 ^b^	-	-	2375.67 ^P–S^
	1	7.12 ± 1.00 ^b^	130.31 ± 9.89 ^de^	186.38 ± 36.64 ^b^	4631.90 ^M–B^	0.638	848.56 ^P–S^
	2	7.01 ± 1.50 ^b^	146.12 ± 11.75 ^e^	183.30 ± 31.86 ^b^	3208.98 ^M–B^	0.574	528.33 ^P–S^
	4	5.18 ± 0.72 ^a^	86.08 ± 39.44 ^bc^	144.11 ± 22.05 ^a^	2330.98 ^M–B^	0.518	258.39 ^P–S^
GEL	0	16.18 ± 0.94 ^d^	56.10 ± 9.02 ^a^	339.72 ± 24.76 ^d^	-	-	1230.76 ^P–S^
	1	16.97 ± 2.24 ^d^	89.79 ± 8.42 ^bc^	230.11 ± 33.24 ^c^	2182.53 ^M–B^	0.626	618.39 ^P–S^
	2	17.43 ± 0.60 ^d^	102.52 ± 11.18 ^c^	226.61 ± 26.29 ^c^	3797.28 ^M–B^	0.644	449.36 ^P–S^
	4	12.92 ± 1.92 ^c^	112.58 ± 24.70 ^cd^	165.33 ± 9.92 ^ab^	2850.30 ^M–B^	0.714	290.77 ^P–S^

^a–e^ Values with the different superscript letters within one column are significantly different (*p* < 0.05). ^M–B^—obtained from the Makoid-Banakar model. ^P–S^—obtained from the Peppas–Sahlin model.

## Data Availability

The raw data supporting the conclusions of this article will be made available by the authors upon request.

## Supplementary Materials

The following supporting information can be downloaded at: https://www.mdpi.com/article/10.3390/polym17030320/s1, Figure S1: A custom-made release-measurement device, consisting of a test tube coupled with a cuvette, used for testing SBO release and antiradical activity; Figure S2: Microscopy images of emulsions made from sodium caseinate (NaCAS) and gelatin (GEL) with increasing concentrations of sea buckthorn oil (SBO); Figure S3: Effect of increasing sea buckthorn oil (SBO) concentrations on UV/VIS light absorbance of sodium caseinate (NaCAS) and gelatin (GEL) films; Data S1: Supplemental Mathematical Modeling SBO Release Data. Data S2: Supplemental Mathematical Modeling DPPH Scavenging Data.
