# Supplementary material for: A Comparative Study on the Structural, Physicochemical, Release, and Antioxidant Properties of Sodium Casein and Gelatin Films Containing Sea Buckthorn Oil
Source: Polymers (Basel). 2025 Jan 24;17(3):320. doi: 10.3390/polym17030320 (PMC11821040; doi:10.3390/polym17030320)
Supplement: Supplementary file 1 [file polymers-17-00320-s001.zip › polymers-3417101-supplementary.pdf]

Article

A Comparative Study on the Structural, Physicochemical, Release, and Antioxidant Properties of Sodium Casein and Gelatin Films Containing Sea Buckthorn Oil

Dariusz Kowalczyk [1,*], Monika Karaś [1], Waldemar Kazimierczak [2], Tomasz Skrzypek [2], Adrian Wiater [3], Artur Bartkowiak [4] and Monika Basiura-Cembala [5]

[1] Department of Biochemistry and Food Chemistry, Faculty of Food Sciences and Biotechnology, University of Life Sciences in Lublin, Skromna 8, 20-704 Lublin, Poland; monika.karas@up.lublin.pl
[2] Department of Biomedicine and Environmental Research, Faculty of Medicine, John Paul II Catholic University of Lublin, Konstantynów 1J, 20-708 Lublin, Poland; waldemar.kazimierczak@kul.pl (W.K.); tomasz.skrzypek@kul.pl (T.S.)
[3] Department of Industrial and Environmental Microbiology, Faculty of Biology and Biotechnology, Maria Curie-Skłodowska University, Akademicka 19, 20-033 Lublin, Poland; adrian.wiater@mail.umcs.pl
[4] Center of Bioimmobilisation and Innovative Packaging Materials, Faculty of Food Sciences and Fisheries, West Pomeranian University of Technology in Szczecin, Klemensa Janickiego 35, 71-270 Szczecin, Poland; artur-bartkowiak@zut.edu.pl
[5] Institute of Engineering Sciences, Faculty of Materials, Civil and Environmental Engineering, University of Bielsko-Biala, Willowa 2, 43-309 Bielsko-Biała, Poland; mbasiura@ubb.edu.pl
* Correspondence: dariusz.kowalczyk@up.lublin.pl

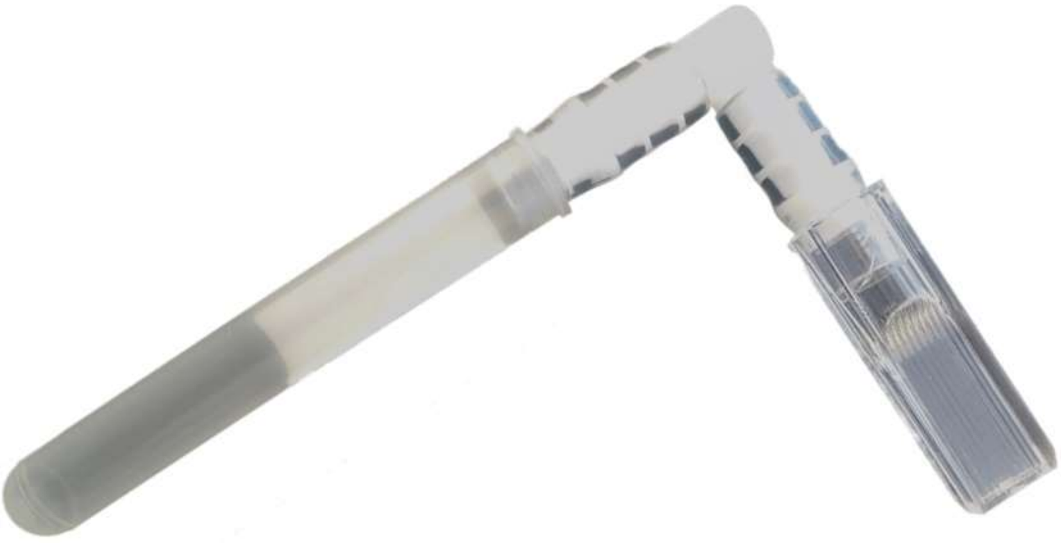

Figure S1. A custom-made release-measurement device, consisting of a test tube coupled with a cuvette, used for testing SBO release and antiradical activity.

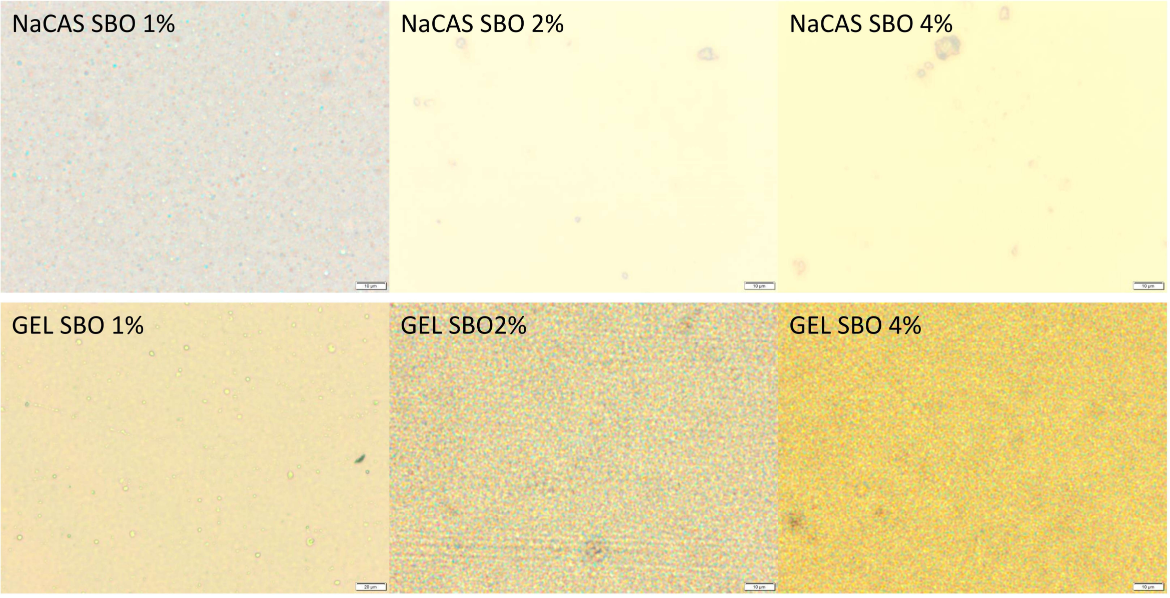

Figure S2. Microscopy images of emulsions made from sodium caseinate (NaCAS) and gelatin (GEL) with increasing concentrations of sea buckthorn oil (SBO).

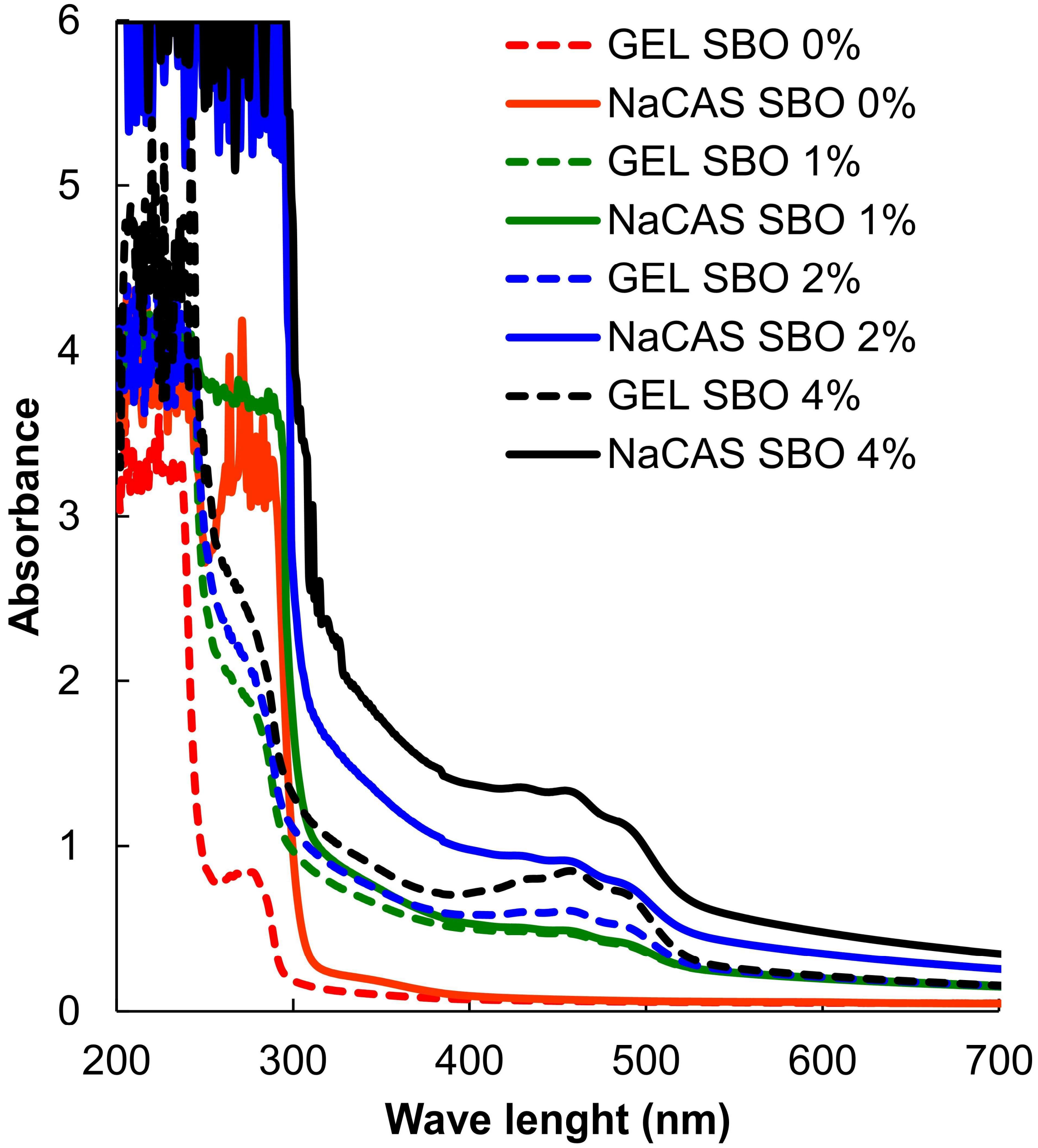

Figure S3. Effect of increasing sea buckthorn oil (SBO) concentrations on UV/VIS light absorbance of sodium caseinate (NaCAS) and gelatin (GEL) films.
